# Identification of force chains in wet coal dust layer and the effect of porosity on three-body contact stiffness

**DOI:** 10.1038/s41598-024-67340-y

**Published:** 2024-07-22

**Authors:** Xinwei Yang, Dongxuan Wu, Yangxi Bai, Hongyue Chen, Xin Wang

**Affiliations:** 1 School of Mechanical Engineering, Liaoning Technical University, Fuxin, 123000 China; 2 Ordos Institute of Liaoning Technical University, Ordos, 017000 China; 3 China National Coal Association, Dynamic Research for High-End Complete Integrated Coal Mining Equipment and Big Data Analysis Centre, Fuxin, 123000 China

**Keywords:** Wet coal dust, Force chain criterion, Three-body contact stiffness, Particle crushing, Engineering, Mechanical engineering, Complex networks, Nonlinear phenomena

## Abstract

Aiming at the three-body contact problem of mechanical rough surface containing wet coal dust interface, the three-body contact model of rough surface containing wet coal dust interface is constructed by comprehensively considering the contact deformation of rough surface and contact characteristics of wet coal dust, and based on the crushing theory. By analysing the contact force, load-bearing particle size and adjacent contact angle thresholds of the wet coal dust layer, the force chain identification criterion is formulated. Finally, quantitative calculations of the force chain characteristics are performed to reveal the effect of different initial porosities on the three-body contact stiffness, which is verified experimentally. The results of the study show that the average contact force of the wet coal dust layer can be used as the force chain contact force threshold, the average particle size can be used as the force chain particle size threshold, and the force chain angle threshold is determined by the particle coordination number. As the initial porosity decreases, the number, length and stiffness of force chains in the wet coal dust layer increase significantly, and the stiffness reaches a maximum value of 2.007 × 10^8^ pa/m at the moment of downward pressure to stabilisation, while the trend of force chain bending varies in the opposite direction, and its minimum bending degree decreases to 20°. The maximum relative error between the simulation and experimental results of three-body contact stiffness is 9.64%, which proves the accuracy of the force chain identification criterion and the quantitative calculation of three-body contact stiffness by force chain.

## Introduction

Under the influence of the working environment of underground coal mines, the coal cutting dust of coal mining machine is mixed with spray water to form wet coal dust. Under the influence of pressure load and shear movement, the wet coal dust will produce "bonding effect" with the bonding surface, and exist in a third body way between the slide-shoe and the guideway bonding surface for a long time^1,2^. Due to the presence of wet coal dust interface, the kinetic instability of the mechanical system is induced, which becomes the main factor restricting the high reliability and stability control of the mechanical system, and there is an urgent need to study the contact characteristics between the bonding surfaces of the coal mine machinery.

At the three-body contact interface, two objects exert a force on the wet coal dust by squeezing each other, and these two objects are called the "first body", while the wet coal dust between them is defined as the "third body". When the granular layer is subjected to external loading a force chain is formed, which is a mesoscale load-bearing bridge through the granular layer and carries the bulk of the external load^3^, and serves as a potential tool for assessing the load-bearing capacity of the granular layer^4,5^. The force chains intertwine to form a network throughout the granular material system and play a decisive role in the macroscopic mechanical properties of the granular system^6^. Therefore, further analysis of the macroscopic mechanical behaviour of the wet coal dust layer in relation to the microscopic interactions between the particles is required to reveal the mechanical properties of the three-body contact bonding surface. This multi-scale mechanical relationship involves micro, meso and macro scales, where previous researchers have characterised the coordination number and contact force distribution of individual particles at the micro scale^7^. On the mesoscopic scale, structural features of force chain evolution and quantification of force chain properties are analyse^8^. On the macroscopic scale, discrete particles are considered as a continuum and a continuum medium model is used to simulate the unique mechanical behaviour of the particulate system^9^. Wang et al^10^. revealed the structural evolution of force chain after local excavation of granular materials by photo-elasticity technique, and further obtained the relationship between the change of force chain properties and the formation of force chain arches by using the G^2^ algorithm, which provided a new perspective and method for solving related geotechnical engineering problems. In a study of continuous loading experiments on a two-dimensional particle system, Chen et al^11^. revealed the variation rules of the deflection angle and coordination number of particles in the force chain over time by digital image correlation (DIC) and found that the structural evolution of the force chain is directly related to the number, geometrical properties and arrangement distribution of particles contacted by the external loading. Huang et al^12^. used PFC 2D to simulate the force chain structure evolution of gangue mixture, and found that most of the force chains are concentrated in the vicinity of larger particle size particles and form the phenomenon of “skeleton force chain”. Initial cracks are mainly generated around the gangue particles, and with the increase of axial pressure, the cracks expand from 30° to 45°, and eventually produce macro cracks. Wang et al^13^. explored the relationship between microscopic parameters and macroscopic mechanical behaviours of granular systems through discrete element simulations and experiments, and found that changes in the force chain distribution and constitutive anisotropy of the granular system were consistent with changes in macroscopic shear strength. Cao et al^14^. used PFC_2D_ and ABAQUS software to establish a discrete element method (DEM) model and a finite element method (FEM)-DEM coupled model to conduct microscopic and macroscopic curved-path force transfer analyses of ultrasonic vibration, and found that ultrasonic vibration coarsened the force chain within the particulate system and increased the efficiency of force transfer. Kang et al^15^. used a continuous-discontinuous numerical method (Numerical Manifold Method (NMM)) to simulate the intrinsic connection between the force chain and particle fragmentation in the particle system, and found out that the particle fragmentation is directly related to the structure of the force chain, the particle fragmentation morphology is basically the same as the direction of the structure of the force chain in the particle system, and the larger particles in the force chain will be slightly fragmented or not fragmented.

To accurately simulate the contact characteristics between the slide-shoe and the guide rail of the coal mining machine, the coupling of finite element and discrete element methods is utilized to comprehensively consider the discrete nature of the particles at the interface of the wet coal dust and the deformation of the contact surface between the slide-shoe and the guide rail. Based on particle fragmentation theory, a three-body contact model containing wet coal dust interface is constructed. Based on the bearing characteristics of the wet coal dust layer, the force chain identification criterion is established and quantitative analysis of the force chain characteristics is carried out, and the effect of different initial porosity on the three-body contact stiffness is revealed and experimentally verified. The results of the study are of guiding significance for the contact characteristics and life analysis of coal mining machine slide-shoe.

## Modelling three-body contact on rough mechanical surfaces

**Figure 1:**
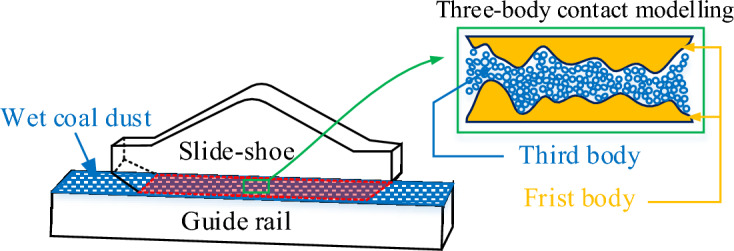
Three-body contact modelling of mechanically rough surfaces.

In the working environment of underground coal mines, wet coal dust will be attached between the slide-shoe and guide rail bonding surfaces of coal mining machines for a long time. Since the particle size of the coal dust and the surface roughness of the machine are in the same order of magnitude, a three-body contact simulation model of the rough surface is established by extracting the surface morphology of the sliding boots and guide rails as well as the physical parameters of the wet coal dust. The slide-shoe and guide rail are the “first body”, and the wet coal dust is the “third body”, as shown in Fig. 1.

### Extraction of mechanical rough surfaces morphology

**Figure 2:**
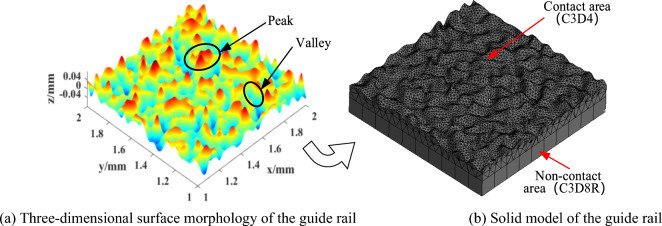
Establishing the solid model of the guide rail.

Almost all mechanical surfaces have a roughness^16,17^, and in practice the surface morphology parameters are different for slide shoes and guideways. The relevant parameters in the fractal function are obtained by AFM measurements as *D*_1_ = 2.3, *G*_1_ = 3.36 × 10^–10^ m for the slide shoe and *D*_2_ = 2.6, *G*_2_ = 3.82 × 10^–10^ m for the guide rail. According to the bivariate W–M function (Eq. 1), the guide rail three-dimensional fractal rough surface topography was generated, as shown in Fig. 2a. The acquired surface topography data points were imported into Creo software to construct the solid model of the guide rail, and the grid was delineated, as shown in Fig. 2b. The peaks and valleys on the surface were randomly distributed, appearing obviously disordered. The guide rail model was built in the same manner as the slide shoe model.

1 $$ z\left( {x,y} \right) = L_{a} \left( {\frac{G}{{L_{a} }}} \right)^{{\left( {D - 2} \right)}} \left( {\frac{\ln \gamma }{M}} \right)^{1/2} \sum\limits_{m = 1}^{M} {\sum\limits_{n = 0}^{{n_{max} }} {\gamma^{{\left( {D - 3} \right)n}} } } \times \left\{ {\cos \varphi_{m,n} - \cos \left[ {\frac{{2\pi \gamma^{n} \left( {x^{2} + y^{2} } \right)^{1/2} }}{{L_{a} }} \times \cos \left( {\tan^{ - 1} \left( \frac{y}{x} \right) - \frac{\pi m}{M}} \right) + \varphi_{m,n} } \right]} \right\} $$

where *z*(*x,y*) is the height of the random contour of the rough surface, *L*_*a*_ is the sampling length, *G* is the fractal roughness, *D* is the fractal dimension (2 < *D* < 3), *M* is the number of directions for generating rough surfaces (*M* = 10), $\varphi$_*m,n*_ are random phases uniformly distributed in (0,2π), *γ* is the frequency density of the generated rough surface (*γ* > 1), and *γ*^*n*^ is the frequency, where n is the frequency exponent, which denotes the rank of the microconvex body^18^. *n*_*max*_ = int[log*(L*_*a*_*/L*_*s*_*)/*logγ], with *Ls* being the cut-off length and *γ* = 1.5.

### Extraction of physical parameters of coal dust

**Figure 3:**
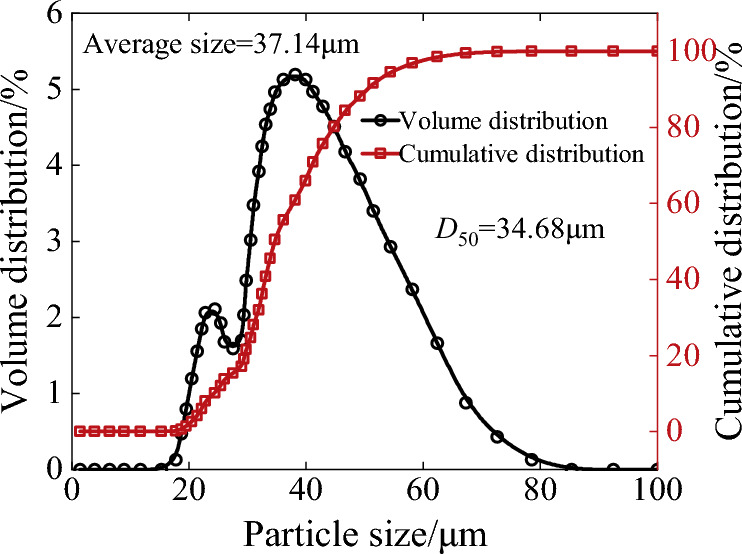
Particle size distribution of wet coal dust.

A sample of coal dust between the coal mining machine slide-shoe and guide rail bonding surface in the comprehensive mining face of a coal mine in northern Shanxi Province was extracted. The moisture content of several groups of extracted coal dust samples was measured, which basically concentrated around more or less 5.4%, with a very small difference. A Malvern laser particle sizer was used to analyse the particle size, and the results are shown in Fig. 3. The particle size volume distribution showed two peaks: the larger particle size coal dust was mixed with some small particle size coal dust, and the mean particle size D_50_ was 34.68 μm. It was relatively difficult to measure the contact parameters between wet coal dust in the test. Therefore, these parameters could be obtained by using a virtual calibration test of the stacking angle^19^. By considering the measured stacking angle of wet coal dust as a reference, the bottomless cylinder model was used for the stacking angle simulation test, and combined with the literature^20^ and generic EDEM material model (GEMM) granular general material database, the parameters of wet coal dust were obtained, as shown in Table 1. The extracted particle size distribution of wet coal dust was imported into the simulation software, and the corresponding contact properties were input to construct the simulated wet coal dust layer. The surface of the particles is actually rough^17^ and uneven in practice, but considering that the particle size of the coal dust particles is very small, the microscopic rough surface of the particles has little effect on the solid–liquid contact angle of the liquid bridge. Therefore, when establishing the moist coal dust layer, the coal dust particles are assumed to be smooth spherical particles.

**Table 1:** Wet coal dust parameters and first body parameters.

Material	Poisson's ratio *υ*	Density *ρ*/kg m^–3^	Elastic modulus *G*/GPa	Material	Coefficient of restitution	Coefficient of static friction	Coefficient of rolling friction
Wet coal	0.34	1400	1.81	Wet coal–wet coal	0.05	0.7	0.1
steel	0.28	8800	180	Wet coal–steel	0.05	0.4	0.1

**Figure 4:**
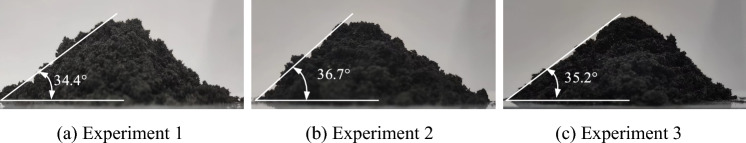
Stacking angle determination.

**Figure 5:**
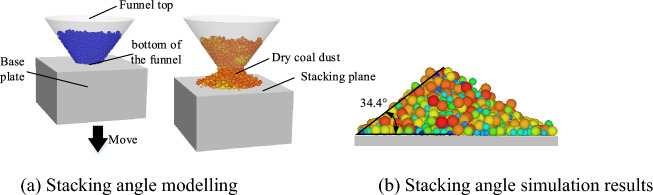
Dry coal dust stacking angle models and simulation results.

Experimental calibration was carried out to obtain accurate physical parameters of coal dust. Firstly, the funnel container with a given mass of dry coal dust was lifted vertically upwards for 10 cm and the dry coal dust particles would move downwards freely. When the stacking angle formed by the fell dry coal dust stabilized, the stacking angle was measured, as shown in Fig. 4. In order to reduce experimental error, three experiments were conducted and the average value (35.43°) was considered as the resultant data. The simulation model using Hertz-Middlin contact model is shown in Fig. 5a. The material differences between the funnel and stacking plane was ignored in the numerical simulation, i.e. they are modeled as the same material. The contact parameters among coal dust particles was adjusted to make the simulation results consistent with the experiment. In order to improve the computational efficiency, the diameter of the funnel mouth and the size of the bottom plate are usually reduced during the simulation resulting in reducing the number of particles involved in the calculation^21^. In addition, the falling height of the particles can be limited by moving the stacking plane upwards, thus reducing the impact and inertia effects of the falling particles^22^. As shown in Fig. 5b, the final angle of repose obtained is 34.4°, which is only 2.91% error from the experimental result.

### Construction of a three-body simulation contact model

**Figure 6:**
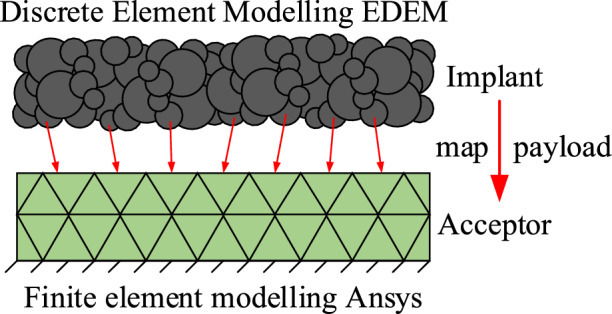
EDEM-ANSYS Workbench coupling principle.

In order to simulate the mechanical properties of the three-body contact surfaces, EDEM-ANSYS Workbench coupling is used in this manuscript, as shown in Fig. 6. Firstly, the solid model is imported into Ansys for meshing and the meshed model is imported into EDEM. Thereby, the discrete and finite elements both use the same mesh type and size, the same global coordinate system is maintained during the coupling process. The particle-geometry contact force and its contact position coordinates were then extracted from the EDEM via a plug-in for EDEM and Ansys coupling. Next, the data transfer modules between EDEM and Ansys were constructed in Ansys. The extracted data were used as input loads in Ansys. Finally, the loads were mapped one by one in Ansys according to the mesh position, from which the effect of wet coal dust on the rail at different moments was obtained to determine the stresses and strains on the rail surface.

**Figure 7:**
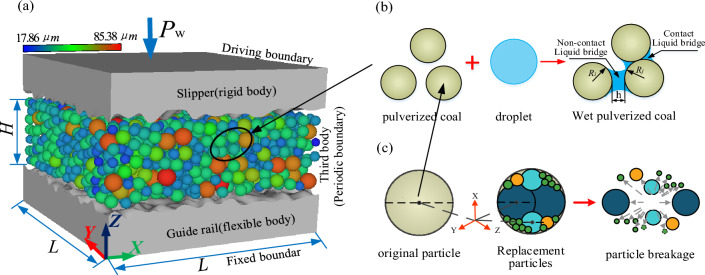
(**a**) Three-body contact (FDEM) model of rough surfaces containing wet coal dust, (**b**) wet coal dust contact model, and (**c**) coal dust particle fragmentation replacement model.

The three-body contact model was constructed, as shown in Fig. 7a, by extracting the surface topographies of the slide- shoe and guide rail and the physical parameters of the wet coal dust. The whole model was divided into two regions: the finite element region of the sliding shoe and guide rail and the discrete element region of the wet coal dust layer. This model could simulate the process of the wet coal dust layer under pressure between the sliding shoe and the guide rail. Initially, the slide-shoe was set as a rigid body, the guide rail was set as a flexible body, and the elastic deformation of the slide-shoe was transferred to the guide rail. The macroscopic gap of the first body was the thickness of the wet coal dust layer *H* = 0.5 mm, the porosity was 0.5, the length of the bottom side of the slide-shoe and guide rail specimen was L = 1 mm, and the periodic boundary was set on the sidewall around the wet coal dust layer. Three-body contact bonding surface area A_L_ = 1mm^2^. In the simulation, the guide rail was fixed and immobile, and the slide-shoe applied normal stress *P*_*w*_ which was combined with the literature values^23^ and multiple simulations of *P*_*w*_ loading was 0.6 Mpa.

### Define the microscopic contact parameters of the wet coal dust layer

In order to accurately simulate the mechanical properties of the three-body contact bonding surface, the wet coal dust microscopic contact model is further defined. The microscopic contact of wet coal dust mainly includes the liquid bridge contact model and the particle crushing contact model, in which the liquid bridge will cause agglomeration of wet coal dust, and the particle crushing when the coal dust particles are of their own nature.

#### Liquid bridge contact model

In addition to the gravitational, frictional, and contact forces, the force on the wet coal dust bonded between the sliding shoe and the guide rail resulted from the liquid bridge force. The liquid bridge between the wet coal dust was divided into a noncontact liquid bridge and a contact liquid bridge, as shown in Fig. 7b, and the adhesion force generated by the liquid bridge was characterized by the custom-developed API liquid bridge model.

In DEM simulations according to the literature^24^, the motion of the particles could be determined by resolving the contact forces generated between neighbouring particles. The trajectory of each particle could be obtained by integrating the Newtonian equations of motion after the initial system configuration, and the motion of each wet pulverized coal particle was determined by the following equations:

2 $$ m_{i} \frac{{dv_{i} }}{dt} = \sum\limits_{j} {\left( {F_{ij}^{N} + F_{ij}^{T} } \right)} + F_{ij}^{c} + m_{i} g $$

3 $$ I_{i} \frac{{d\omega_{i} }}{dt} = \sum\limits_{j} {\left( {R_{i} \times F_{ij}^{T} } \right)} + \tau_{rij} $$

where *m*_*i*_, *Ri*, *I*_*i*_, *V*_*i*_, and *ω*_*i*_ are the mass, radius, moment of inertia, linear and angular velocities of particle *i*, respectively; *g* is the acceleration due to gravity; *FN ij* and *FT ij* are the normal and tangential forces, respectively, generated by the contact of particle *i* with particle *j*; and *F*c ij is the cohesive force that forms a liquid bridge between particle *i* and particle *j*.

Based on the liquid bridge theory of Mikami^25^, the liquid bridge adhesion and the liquid bridge volume were related to the particle spacing as an explicit function:

4 $$ \hat{F}^{{\text{c}}} = \exp \left( {Ah^{*} + B} \right) + C $$

where $\hat{F}^{{\text{c}}}$ is the dimensionless interparticle liquid bridge force ($\hat{F}^{{\text{c}}}$ = *F*_*c*_/2πγR^*^), *γ* is the liquid surface tension, parameters *A*, *B* and *C* are functions of the liquid bridge volume *V* and the particle radius, h^*^ is the dimensionless two-particle surface spacing coefficient (h^*^ = h/R^*^), *h* is the distance between the two particle surfaces, and R^*^ is the equivalent radius.

The forces between the wet coal dust particles were defined as follows:

5 $$ \begin{gathered} A = - 1.1(V^{*} )^{ - 0.53} \hfill \\ B = \left( { - 0.34\ln V^{*} - 0.96} \right)\theta^{2} - 0.019\ln V^{*} + 0.48 \hfill \\ C = 0.0042\ln V^{*} + 0.078 \hfill \\ \end{gathered} $$

The forces between the wet coal dust and the slide shoe (guide rail) were as follows:

6 $$ \begin{gathered} A = - 1.9(V^{*} )^{ - 0.51} \hfill \\ B = \left( { - 0.016\ln V^{*} - 0.76} \right)\theta^{2} - 0.12\ln V^{*} + 1.2 \hfill \\ C = 0.013\ln V^{*} + 0.18 \hfill \\ \end{gathered} $$

where *θ* is the solid‒liquid contact angle, V^*^ is the dimensionless liquid bridge volume (V^*^ = V/R^*3^), and *V* is the total volume of the liquid bridge between particles.

The liquid bridge volume *V* was calculated based on the analysis of McCarthy et al^26^:

7 $$ V = V_{i} + V_{j} $$

8 $$ V_{i} = \frac{{SM_{i} }}{2\rho } \times \left( {1 - \sqrt {1 - {{R_{j}^{2} } \mathord{\left/ {\vphantom {{R_{j}^{2} } {(R_{i} + R_{j} )^{2} }}} \right. \kern-0pt} {(R_{i} + R_{j} )^{2} }}} } \right) $$

where *V*_*i*_ and *V*_*j*_ are the liquid bridge volumes on particles *i* and *j*, respectively; *S* is the extrinsic water content of particle *i*; *M*_*i*_ is the mass of particle *i*; and *ρ* is the density of the liquid.

When the distance between two objects forming a bridge increased, the bridge underwent tensile deformation. When the distance was greater than a specific value of the bridge breaks, the bridge breakage limited both the distance, the bridge volume and the solid‒liquid contact angle. The particle-liquid bridge contact parameters are shown in Table 2.

**Table 2:** Particle liquid bridge and crushing parameters.

Liquid bridge parameter	Value
Liquid	Water
Surface tension/(N m^–1^)	0.0728
Contact angle/rad	0
Density *ρ*/(kg m^–3^)	1000
Viscosity/pa s	2.98 × 10^–3^

The liquid bridge limit distance *S*_*cp*_ between wet pulverized coal particles was as follows:

9 $$ {\text{s}}_{cp} /R^{ * } = (0.62\theta + 0.99)(V^{*} )^{0.34} $$

The limit distance *S*_*cw*_ between the wet coal dust and slide shoe (guide rail) was as follows:

10 $$ s_{{c{\text{w}}}} /R^{ * } = (0.22\theta + 0.95)(V^{*} )^{0.32} $$

11 $$ \frac{1}{{R^{ * } }} = \frac{1}{2}\left( {\frac{1}{{R_{i} }} + \frac{1}{{R_{j} }}} \right) $$

where *R*_*i*_ and *R*_*j*_ are the radii of the two particles forming the liquid bridge, and the radius of the liquid bridge between the particles and the upper guide is equal to the radius of the particles.

#### Particle crushing replacement model

In the actual contact process, the plastic deformation of the wet coal dust could be very small and instantly break^27^. Figure 7c demonstrates the particle replacement process. To simulate realistic crushing results, small subparticles were concentrated inside the parent particle in the region of applied stress, while the large subparticles were aligned perpendicular to the direction of the rupture stress; the rest of the subparticles filled the voids of the parent particle^28,29^. During particle replacement, subparticles were allowed to undergo an initial overlap to accommodate the volume of the parent particle. To avoid explosions caused by large repulsive forces due to overlap, damping was used to limit the total contact force applied to each subparticle and to reduce the duration of the force. In addition, based on Griffith's theory^30^, the amount of overlap between subparticles was controlled to take into account the energy dissipation during internal fracture and to prevent extremely high velocities of subparticles during fragmentation.

**Figure 8:**
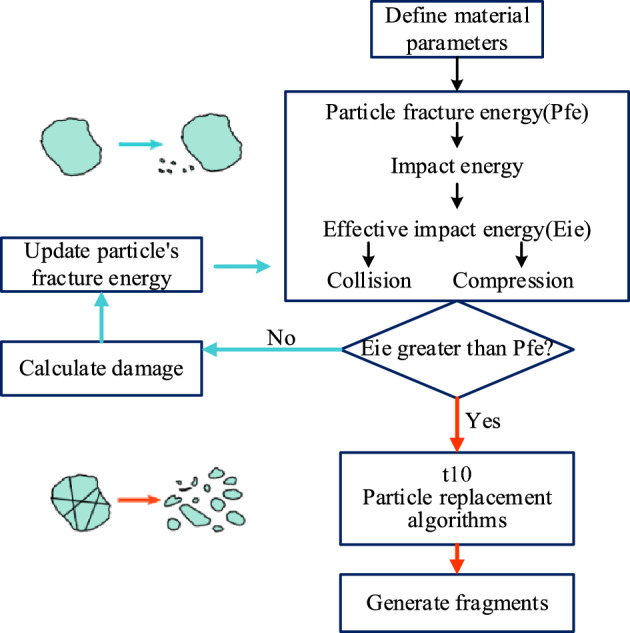
Particle crushing process.

The wet coal dust particle crushing procedure is shown in Fig. 8. When the effective impact energy of the particles was less than the crushing energy, due to particle movement, collision wear could produce surface crushing. When the effective impact energy was greater than the particle fracture energy, fracture occurred within the particle. The particles could be broken several times, and the broken subparticles were broken again in accordance with the crushing program. Each particle had a specific fracture energy, which was assigned based on its size, mean and standard deviation values. This energy could vary according to the upper truncated lognormal distribution^31^, which was defined as follows:

12 $$ P(E) = \frac{1}{2}\left[ {1 + erf\frac{{\ln E* - \ln E_{50} }}{\sqrt 2 \sigma }} \right] $$

13 $$ E^{*} = \frac{{E_{max} E}}{{E_{max} - E}} $$

where *E* is the particle fracture energy distribution corresponding to the maximum stress energy that the particle can withstand in a collision, *E*_*max*_ is the upper truncated value of the distribution, and *E*_*50*_ and *σ* are the median and standard deviation of the distribution, respectively. The median specific fracture energy of a particle was defined as follows:

14 $$ E_{50} = \frac{{E_{\infty } }}{{1 + k_{p} /k_{s} }}\left[ {1 + (\frac{{d_{0} }}{{d_{p} }})^{\varphi } } \right] $$

where *E*_*∞*_, *d*_*o*_ and *φ* are model parameters that must be fitted to the experimental data, *d*_*p*_ is the representative grain size included in the grain size hierarchy.

The surface of the particle without fracture could suffer wear, and the particle could produce a new fracture energy^36^ with the following value:

15 $$ E_{f}^{\prime } = E_{f} (1 - D) $$

16 $$ D = \left[ {\frac{{2\eta eE_{k} }}{{(2\eta - 5D + 5)E_{f} }}} \right]^{{\frac{2\eta }{5}}} $$

where *E*_*f*_ is the fracture energy of the particle, *D* is the breakage, *eE*_*k*_ is the effective impact energy in the collision, *η* is the breakage accumulation factor, and *e* is the proportion of energy involved in the collision, which was assigned to the particle according to its stiffness.

17 $$ e = \frac{1}{{\left( {1 + k_{p} /k_{s} } \right)}} $$

where *k*_*p*_ is the particle stiffness and *k*_*s*_ is the particle surface stiffness. Two particles of the same shape collided with an average energy distribution of *e* = 0.5.

The degree of particle fragmentation was represented by a single parameter, t_10_, reflecting the proportion of subparticles smaller than 1/10 of the parent particle size^28,29^. The degree of fragmentation when stress was applied to multiple particles was related to the particle-specific stress energy and the median fracture energy^32^ with the following expression:

18 $$ t_{10} = A\left[ {1 - exp \left( { - b\frac{{eE_{K} }}{{E_{f} }}} \right)} \right] $$

where *A* and *b* are impact parameters and *A* corresponds to the maximum value of *t*_10_ that can be achieved when crushing material in a single impact. The higher the specific impact energy *E*_*k*_ was, the larger the *t*_10_ value and the finer the size distribution of the broken subparticles compared to the specific fracture energy of the particles.

**Figure 9:**
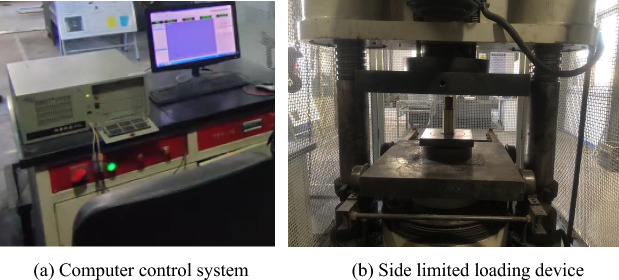
YAW-2000 Testing Machine.

In order to get the accurate crushing parameters of coal powder particles, YAW-2000 microcomputer-controlled electro-hydraulic servo pressure tester was used, and the wet coal powder particles were added into the autoclave body with an inner diameter of 32 mm and an outer diameter of 38 mm in Fig. 9b, as shown in Fig. 9. The indenter was placed on top of the wet coal dust, and the downward pressure speed of the loading plate was controlled to be 0.1 mm/s. The stress–strain curve of the wet coal dust specimen was recorded in real time. In this paper, we refer to the calibrated values of coal mine crushing parameters of Tavares^33,34^ and calibrate the crushing parameters of humid coal dust, and finally obtain the crushing parameters of simulated humid coal dust as shown in Table 3.

**Table 3:** Particle crushing parameters.

Parameter	Symbol	Value
Damage constant		3
Fitting parameter	φ	1.28
E_50_ (mm)	*d*_0_	4.3
E_50_ (J/kg)	*E*_∞_	44.9
Standard deviation of the fracture energy	σ_E_	0.46
Minimum collision energy (J)	*E*_min_	0.0001
Minimum particle size for breakage (mm)	*d*_min_	0.012
Impact breakage parameter (%)	A	60.4
Impact breakage parameter	b	0.051

**Figure 10:**
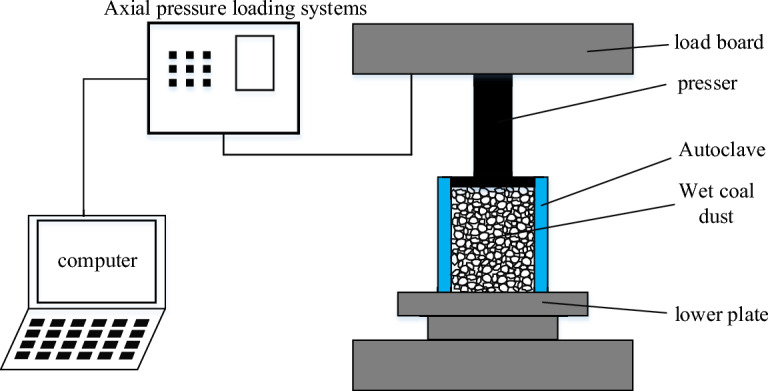
Side-limited loading test system.

As shown in Fig. 10, this paper establishes the same side-limit loading simulation model as that of the test, and continuously adjusts the simulation crushing parameters by comparing the error values of the simulation and test stress–strain curves until they are consistent with the changes of the stress–strain curves obtained from the test. In the side-limit loading test, the wet coal dust layer successively experienced the initial stage, slip stage, crushing stage (multi-stage crushing) and compaction stage.

**Figure 11:**
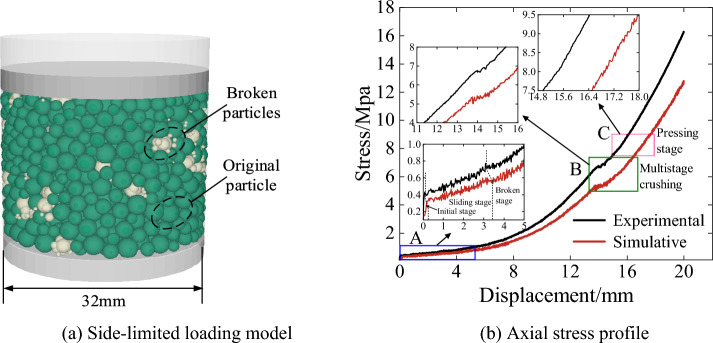
Model and results of Side-limited loading test.

The simulated limit-side compression model constructed in this paper is shown in Fig. 11a. In the initial stage, the stress increases mainly linearly and increases to about 0.4 MPa as the porosity decreases between the loading plate under the loading of the loose wet coal dust; in the slip stage, the stress increases and produces small fluctuations up and down, which is mainly due to the rearrangement of the particle position under the external load and a small number of particles are crushed in this stage, as shown in A in Fig. 11b. In the crushing stage, a large number of particles are crushed by the close contact between wet coal dust, and the stress is in the up and down fluctuation change. And as the external load continues to increase, a large number of particles occurred several times broken, the kinetic energy input to the system at this stage is mainly consumed by particle crushing and friction, as shown in B in Fig. 11b. In the compression-solidification stage, which occurs at the late stage of loading, the broken small particles at this stage fill the voids created by the contact of the large particles. The stress–strain curve rises smoothly without fluctuation and macroscopically shows strain hardening, and most of the external energy in this stage is stored in the wet coal dust^35^, as shown in C in Fig. 11b. In the simulation, small fluctuations in the stress–strain curve due to inter-particle micro-movement occur during the compaction stage. Although the wet coal dust layer cannot reach strain hardening in the simulation, it cannot make the wet coal dust layer compressed into bonding into hard lumps. However, this paper mainly calibrates the parameters of coal dust particle crushing based on the initial stage, slip stage and crushing stage before C point. Therefore, the large error of the compaction stage after point C has no effect on the study of this paper.

### Stress transfer law of wet coal dust layer

**Figure 12:**
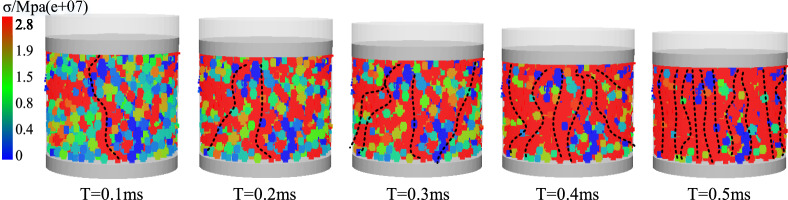
Normal stress cloud of wet coal dust layer.

Through the side-limit loading test, the stress transfer law of the wet coal dust layer is preliminarily analysed, as shown in Fig. 12 Under the action of external load, there are some chain-like structures inside the wet coal dust layer which are thicker and subject to larger stress.

These structures extend from the top to the bottom and play an important role in transferring the stresses. With the increase of loading time, a large number of force chain structures were generated in the wet coal dust layer at T = 0.5 ms to transfer the load. This phenomenon is consistent with the force chain theory in the mechanics of granular materials, which further confirms the feasibility of using mesoscopic force chains to analyse the load-bearing characteristics of the wet coal dust layer.

In this paper, the force chain formation criterion of wet coal dust layer is constructed, the correlation between microscopic contact properties and macroscopic stiffness is established through mesoscopic force chain, and the quantitative analysis of force chain properties is carried out, so as to analyse the influence of different initial porosity on three-body contact stiffness.

## Development of guidelines for force chaining of wet coal dust layer

The force chain formation criterion of the particle system generally includes three conditions^34^: (1) the number of particles constituting the force chain; (2) the contact force criterion of the force chain formation; and (3) the angular threshold criterion of the force chain formation; however, in this paper, the coal particles will be crushed, and the particles of different particle sizes are loaded differently. Therefore, a fourth condition is needed, i.e., (4) force chain formation particle size criterion.

### Force chain formation contact force guidelines

**Figure 13:**
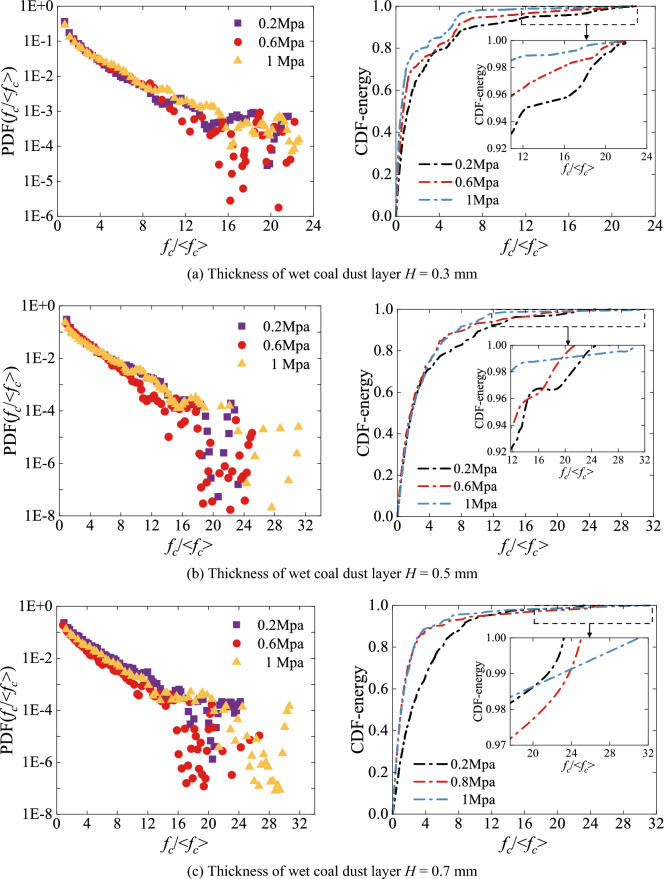
Distribution of contact force and cumulative elastic energy for different thicknesses of wet coal dust layers (*H* = 0.3, 0.5, 0.7 mm).

The wet coal dust layer between the three-body contact bonding surfaces forms a force chain network to transfer loads under external loads, and the current research on the force chain structure of granular materials lacks a unified definition standard, especially involving few guidelines for screening the force chain of granular crushing. Existing studies are mainly related to the contact force, the extension direction of force chain particles and the number of particles in the force chain. Due to the “grading effect” of particle crushing in wet coal beds, the applicability of the traditional chain-forming criteria to the study of force chains in wet coal beds needs to be further verified. By applying different preload forces to the slide-shoe, the EDEM wet coal dust layer contact force and its corresponding elastic strain energy data were extracted, and the distribution of the contact force and the cumulative contact elastic strain energy were analysed by writing a program in Matlab, as shown in Fig. 13. In the figure < *f*_*c*_ > denotes the average contact force of the wet coal dust layer and *f*_*c*_/ < *f*_*c*_ > is used to denote the normalisation of the corresponding contact force. The contact force and elastic strain energy between two-phase contacting particles *R*_1_ and *R*_2_ are:

19 $$ f_{c} = \frac{4}{3}E*(R*)^{{{1 \mathord{\left/ {\vphantom {1 2}} \right. \kern-0pt} 2}}} \delta_{p}^{{{3 \mathord{\left/ {\vphantom {3 2}} \right. \kern-0pt} 2}}} $$

20 $$ W \approx \frac{1}{5R*}\left( {\frac{3R*}{{2E^{\varepsilon } }}\left( {1 - \nu^{2} } \right)} \right)^{{{2 \mathord{\left/ {\vphantom {2 3}} \right. \kern-0pt} 3}}} f_{c}^{{{5 \mathord{\left/ {\vphantom {5 3}} \right. \kern-0pt} 3}}} $$

where 1/*E*^ε^ = (1 − *ν*_1_^2^)/*E*_1_ + (1 − *ν*_2_^2^)/*E*_2_, *E*_1_, *E*_2_, *ν*_1_, *ν*_2_ are the Young's modulus and Poisson's ratio of the contacting particle 1 and particle 2, respectively, and *δ*_*p*_ is the amount of contact overlap (elastic deformation) of the two particles.

Average contact force of wet coal dust layer < *f*_*c*_ > :

21 $$ \left\langle {f_{c} } \right\rangle = \frac{{\sum {f_{c} } }}{{N_{P} }} $$

where *N*_*P*_ is the total number of particle contacts, Σ*f*_*c*_ is the sum of particle contact forces within the model.

It can be seen from the results in Fig. 13 that, with the increase of contact force, the proportion of contacts in the wet coal dust layer firstly decreases linearly, and then the distribution becomes chaotic and irregular, and the proportion of contacts with smaller contact force is larger. At the same time, the contact elastic strain energy of the whole humid coal dust layer increases with the contact force. The elastic strain energy first increases proportionally and cumulatively, and then slowly increases to 1 with a slowing trend. By comparing the cumulative elastic energy of different thicknesses of the wet coal dust layer, it can be seen that the range of normalised *f*/ < *f*_*c*_ > of the contact force within the wet coal dust layer increases with the increase of the thickness of the layer, from 22 to about 32. This suggests that the wet coal dust layer with larger thickness is decomposed into more different force intervals in the process of transferring the external load applied by the sliding shoe to the guideway surface, inducing the formation of a force chain network with different structures in different regions of the wet coal dust layer. In addition, the elastic cumulative energy of the wet coal dust layer with larger thickness preferentially reaches a levelling off trend due to the increase in the number of load-bearing particles.

As can be seen from Fig. 13, for a wet coal dust layer with a thickness of 0.3 mm, the proportion of the number of contact points smaller than the average contact force is about 70% to 75%, while the corresponding proportion of the total elastic strain energy is only about 6.5–12%. In contrast, the number of contact points larger than the average contact force is about 26%, but the proportion of total elastic strain energy is as high as about 87–93%. For 0.5 mm thick wet coal seam, the number of contact points smaller than the average contact force is about 70%, while the corresponding proportion of total elastic strain energy is only about 9%. In contrast, the number of contact points larger than the average contact force is about 28%, but the proportion of total elastic strain energy is as high as about 90%.

For a wet coal dust layer with a thickness of 0.7 mm, the number of contact points smaller than the average contact force is about 73%, while the corresponding proportion of total elastic strain energy is only about 9%. Similarly, the number of contact points larger than the average contact force is about 26–30%, but the proportion of total elastic strain energy is as high as about 90%.

Based on the results of the above analyses, it can be seen that the percentage of the number of contact points smaller than the average contact force increases slightly with the increase of the thickness of the wet coal dust layer, but the contribution to the total elastic strain energy is relatively small. On the contrary, the number of contact points larger than the average contact force is relatively small, but the contribution to the total elastic strain energy is very significant. Therefore, the contact force greater than the average contact force is used as one of the criteria for force chain formation in the chain formation criteria of wet coal dust layer.

#### Validation of chain-forming contact force guideline

**Figure 14:**
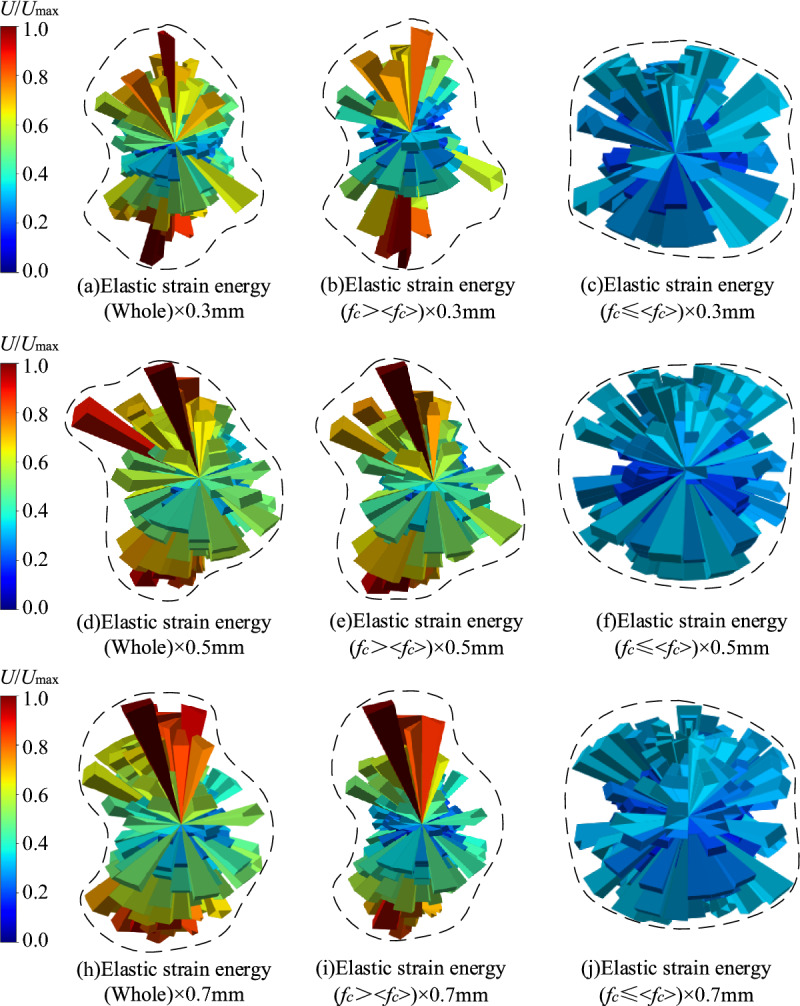
Elastic strain energy distribution of wet coal dust layer with different thicknesses (*H* = 0.3, 0.5, 0.7 mm).

In order to further verify the reliability of the chain-forming contact force criterion, the elastic strain energy distributions of different thicknesses of the wet coal dust layer were analysed separately and compared with those greater than and less than the average contact force. As shown in Fig. 14, in which the elastic strain energy distribution of *f*_*c*_ >  < *f*_*c*_ > is consistent with the elastic strain energy distribution of the wet coal dust layer as a whole, both of them show the anisotropy of the elastic strain energy distribution. This indicates that the elastic strain energy can well reflect the mechanical behaviour of the interior of the wet coal dust layer under loading when the contact force is larger than the average contact force. As for the case of *f*_*c*_ ≤  < *f*_*c*_ > , it can be seen from the figure that the elastic strain energy distribution exhibits an approximately isotropic distribution characteristic, which fails to reflect the loading under load. From the point of view of the elastic strain energy distribution, contacts greater than and less than the average contact force can be regarded as two essentially different contact modes. In particular, the contact greater than the average contact force is directly related to the macroscopic nature of the wet coal dust layer. Therefore, the contact force greater than the average contact force is taken as one of the criteria for the formation of the force chain of the wet coal dust layer.

### Chain-forming particle size guidelines

Under the action of external load, the wet coal dust particles will be broken, and this breaking can happen at any moment during the downward pressure process. Therefore, in order to accurately and reliably extract the force chain structure in the wet coal dust layer, it is necessary to consider different preload forces and different coal dust layer thicknesses at the same time. This enables a more comprehensive understanding of the mechanical properties of the wet coal fly ash layer under external loading, and further analyses of the force chain extraction procedure based on its mechanical properties in order to obtain more accurate results.

**Figure 15:**
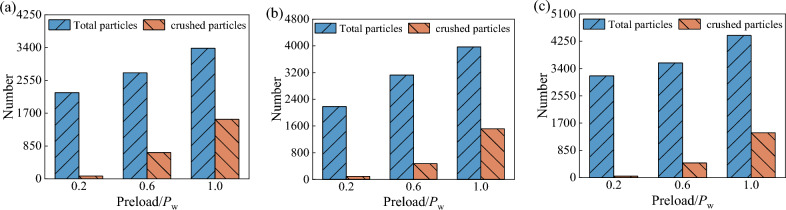
The total number of particles and the number of broken particles in the wet coal dust layer with different thicknesses, (**a**) the thickness of coal layer *H* = 0.3 mm, (**b**) the thickness of coal layer *H* = 0.5 mm, (**c**) the thickness of coal layer *H* = 0.7 mm.

Figure 15 demonstrates the variation of the total number of particles and the number of broken particles in the wet coal dust layer under different preload forces. From the figure, it can be seen that the number of broken particles tends to increase and then decrease with the increase of the thickness of the wet coal dust layer, and the largest number of broken particles occurred when the thickness of the wet coal dust layer was 0.5 mm.

**Figure 16:**
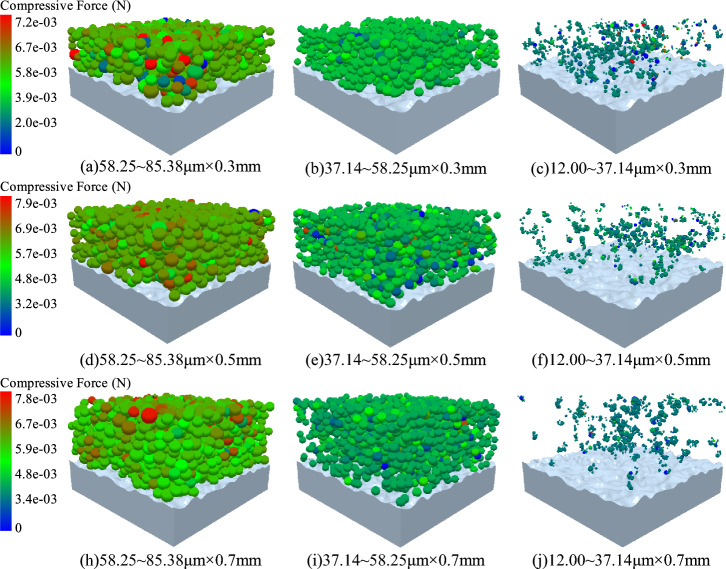
Characteristics of bearing particle size distribution of different thicknesses of wet coal dust layer (*H* = 0.3, 0.5, 0.7 mm).

The different particle size bearing characteristics of the wet coal dust layer after crushing were further analysed, as shown in Fig. 16. By taking the average particle size of the wet coal dust layer as the boundary, the particle size of the wet coal dust layer is mainly divided into three regions, which are 12.00–37.14 μm, 37.14–58.25 μm, and 58.25–85.38 μm, respectively, and the different particle sizes of the internal bearing force of different thicknesses of the wet coal dust layer under the preloading force (*P*_*w*_ = 1 Mpa) are analysed. It can be observed from (a), (d), and (h) in the figure that inside the wet coal dust layer, the large-size particles are numerous and densely distributed to play the main load-bearing role; whereas (b), (e), and (i) have a large number of particle sizes but some of the particles are distributed dispersed and play a secondary load-bearing role. In addition, (c), (f), and (j) in the figure indicate the sub-particles formed by the crushing of the wet coal dust particles, and the distribution of the crushed coal dust particles is scattered with small bearing capacity, and only a small portion of the particles are subjected to the average force. According to the preliminary analysis of the distribution of different particle sizes and forces in the whole wet coal dust layer, it can be obtained that the broken sub-particles fill the voids formed by the contact of large particles, and this phenomenon is reflected in different thicknesses of the wet coal dust layer.

**Figure 17:**
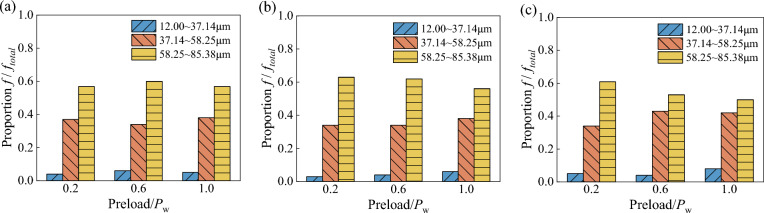
The proportion of particle size of wet coal layer with different thickness, (**a**) the thickness of wet coal layer *H* = 0.3 mm, (**b**) the thickness of wet coal layer *H* = 0.5 mm, (**c**) the thickness of wet coal layer *H* = 0.7 mm.

The small particles broken in the wet coal dust layer will seriously affect the retrieval efficiency of the chain, and for this reason, the different particle sizes of the wet coal dust layer are further analysed for the load-bearing situation. As shown in Fig. 17, the bearing percentage of each particle size region is quantified, which is derived from the ratio of the force on the particles in the particle size region and the overall force. From the figure, it can be clearly seen that the internal particle sizes of different thicknesses of the wet coal dust layer show an obvious carrying pattern, with large particle size carrying ratio > medium particle size carrying ratio > small particle size carrying ratio. The sum of large-size and medium-size bearing ratios is nearly greater than 0.9, which indicates that the medium-size and large-size particles play the main bearing role after the particles in the wet coal seam are broken^36^.

From the above analysis and in conjunction with Fig. 16, it can be seen that the small particles in the wet coal dust layer escape the force transfer by filling the gaps between the large particles. As the gap between the large particles decreases, only a small part of the small particles will be squeezed and transfer the load, and the carrying ratio is not more than 0.5. Therefore, the small particles are not the main skeleton for transferring the load. In addition, special attention should be paid to the fact that when the particles in the wet coal dust layer are broken several times, the "gradation effect" in the whole layer becomes obvious, i.e., the difference in the particle size of small and large particles increases significantly, and the contact force within the group of small particles is very complicated. Therefore, when considering the criteria for the formation of force chains in a wet coal dust layer, it is necessary to take into account the particle size of the particles that form the force chains, and to screen the force chains only for wet coal dust that is larger than the average particle size.

The results of the above analyses show that particle fragmentation in the wet coal layer produces a “grading effect”, which changes the object of the particles forming the force chain. These force chain network structures are mainly carried by larger particles (particles larger than the average particle size of 37.14 μm), while the broken small particles mainly fill the voids. Therefore, when considering the criteria for chain formation in wet coal seams, particle size larger than the average particle size should be taken as one of the criteria for chain formation without affecting the accuracy of chain retrieval and at the same time improving the retrieval efficiency.

### Threshold guidelines for force chain formation angle

**Figure 18:**
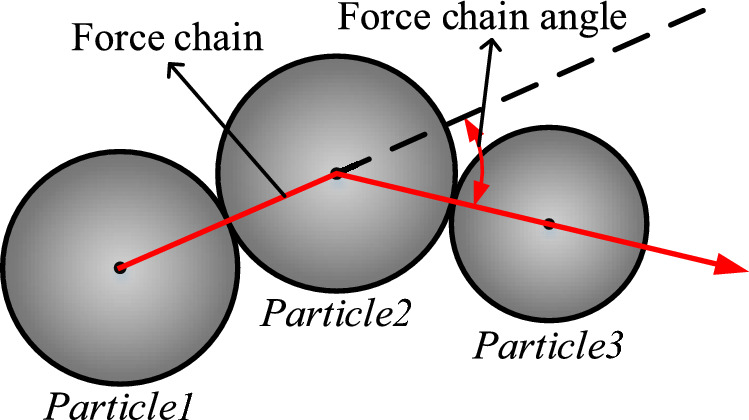
Schematic diagram of force chain angles.

Most researchers generally agree that force chains should be collinear and linear in transmitting stresses, however, how to accurately define the collinearity of force chains is still an unsolved challenge. Currently, in order to control the collinearity of the force chain, the force chain angle threshold *θ*_*thd*_ is usually used to define it, as shown in Fig. 18. However, different scholars have different understandings of the chain-forming angle threshold criterion, so there is no widely accepted definition. An increase in the force chain angle threshold *θ*_*thd*_ increases the range of screening force chains, which leads to an increase in the length and number of force chains in the particulate system. In contrast, a decrease in the force chain angle threshold *θ*_*thd*_ results in a decrease in the length and number of force chains in the particulate system. Different researchers have taken different values for *θ*_*thd*_, Peters et al^37^. took *θ*_*thd*_ to be 45°, while Pschel et al^38^. took *θ*_*thd*_ to be 30°, and Professor Qicheng Sun^39^ defined the value of *θ*_*thd*_ in terms of the average number of collocations, and provided further justification for the value of *θ*_*thd*_. These three types of metrics are representative values of force chain angle thresholds, but none of them involves the particle system where fragmentation can occur.

**Figure 19:**
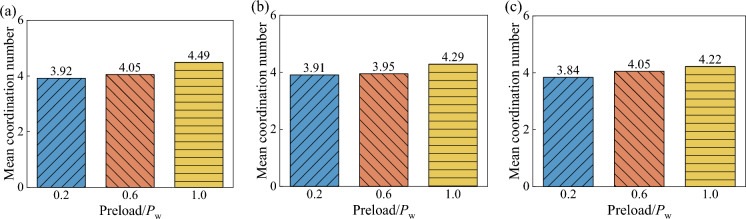
Mean coordination number for different thicknesses of wet coal dust layer, (**a**) the thickness of wet coal layer *H* = 0.3 mm, (**b**) the thickness of wet coal layer *H* = 0.5 mm, (**c**) the thickness of wet coal layer *H* = 0.7 mm.

In this paper, in conjunction with Professor Sun Qicheng's principle of using the definition of coordination number for the force chain threshold, it is considered that small particles undergoing fragmentation in the wet coal seam will lead to an increase in the gradation effect, and therefore further analysis of the threshold criterion for the force chain formation angle of the wet coal dust layer is required. As shown in Fig. 19, the average allotment number of the wet coal dust layer was counted. From the figure, it can be seen that the average coordination number of the wet coal dust layer increases with the increase of the preload force, and also increases with the increase of the thickness of the coal dust layer. This indicates that the broken particles change the coordination number of each particle in the wet coal dust layer, thus changing the structure of the load-bearing force chain. Also according to the previously proposed force chain contact force criterion and particle size criterion on the basis of the analysis, it can be seen that the wet coal dust layer under the action of external loads to form a large number of compression contact, these compression contact force will lead to the wet coal dust particles broken. The small particles generated by the crushing will change the coordination number of the wet coal dust layer, resulting in the change of the structure of the load-bearing force chain.

In this paper, taking into account the “grading effect” generated by the particle crushing, the angle threshold of each force chain is defined according to the size of the coordination number of each particle, and the angle threshold of the force chain is defined as

22 $$ \theta_{thd1} = \frac{\pi }{c} $$

where *c* is the coordination number of individual particles, *c* ≥ 1。

In order to obtain reliable and accurate chain-forming angle thresholds for force chains in the wet coal seam, different angle thresholds were compared and analysed, and their effectiveness was assessed by indicators such as the number of force chains. In this paper, four types of angle thresholds are defined, which are the first type of angle threshold *θ*_*thd1*_ (set to 45°), the second type of angle threshold *θ*_*thd2*_ (set to 30°), the third type of angle threshold *θ*_*thd3*_, which is proposed by Prof Qicheng Sun (*θ*_*thd3*_ = 180/ < c > , and < c > is the average number of alignments of the wet coal dust layer), and the fourth type of angle threshold is the fourth type of angle threshold, which is proposed by this paper, *θ*_*thd4*_.

**Figure 20:**
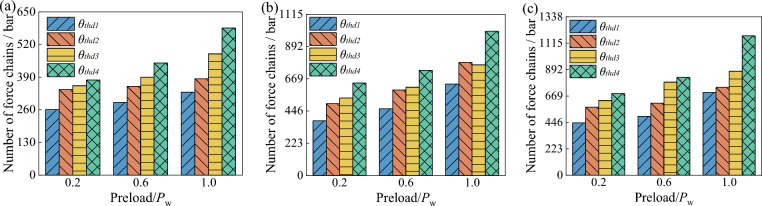
Force chain number of wet coal layer with different thickness, (**a**) the thickness of wet coal layer *H* = 0.3 mm, (**b**) the thickness of wet coal layer *H* = 0.5 mm, (**c**) the thickness of wet coal layer *H* = 0.7 mm.

**Figure 21:**
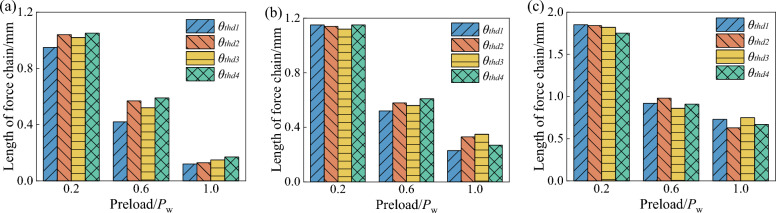
Force chain length of wet coal layer with different thickness, (**a**) the thickness of wet coal layer *H* = 0.3 mm, (**b**) the thickness of wet coal layer *H* = 0.5 mm, (**c**) the thickness of wet coal layer *H* = 0.7 mm.

From Fig. 20, it can be seen that the number of force chains in the wet coal dust layer increases with the increase of preload force and coal dust layer thickness, and all four types of angle thresholds show the same pattern. The larger the force chain angle threshold, the larger the force chain retrieval range increases the probability of chain formation. Further, the force chain angle thresholds were compared by wetting the average force chain force chain length of the coal dust layer. As shown in Fig. 21, the average force chain length of the wet coal dust layer is the distance between the particle centre of mass coordinates of the force chain and divided by the total number of force chains. From the figure, it can be seen that the average length of force chain of wet coal dust layer decreases with the increase of preload force and increases with the increase of the thickness of coal dust layer, and the four types of angular thresholds are in line with this law, but the different types of angular thresholds have less influence on the average length of force chain of wet coal dust layer.

The results of the above analyses show that different types of force chain formation angle thresholds have less influence on the average force chain length of the wet coal dust layer and more influence on the number of force chains. The fourth type of angle threshold defined in this paper retrieves the largest number of force chain force chains, and the least constraint on the force chain angle threshold best reflects the internal contact characteristics of the wet coal dust layer.

### Development of a force-chaining search programme

The wet coal dust bonded between the three-body contact bonding surfaces is not a continuous medium, and the external loads applied to the slide shoe are transferred to the guide through interparticle contact (force chain). Combined with the previous analysis of the criteria required for the force chain, we obtained three criteria for the force chain to form a chain^40^: (1) contact force criterion: the contact force is greater than the average contact force of the wet coal bed (*f*_*c*_ >  < *f*_*c*_ >); (2) particle size criterion: the particle size of the force chain is greater than the average particle size (*D* > 37.14 μm); (3) length criterion: the number of particles in the formation of the force chain is not less than three particles; (4) angular threshold of the force chain criterion: the angle between the two adjacent normal contacts of the force chain must be less than the angle threshold *θ*_*thd*_, so as to ensure the reliability of the force chain, the angle threshold *θ*_*thd*_ = *π*/*c*.

23 $$ (C) = \left( {\arccos \frac{{a^{2} + b^{2} - c^{2} }}{2ab}} \right)\frac{180}{\pi } $$

where (*c*) is the force chain angle, *a* is the distance between particle 1 and particle 2 centre of mass, *b* is the distance between particle 1 and particle 3 centre of mass, and *c* is the distance between particle 2 and particle 3 centre of mass.

**Figure 22:**
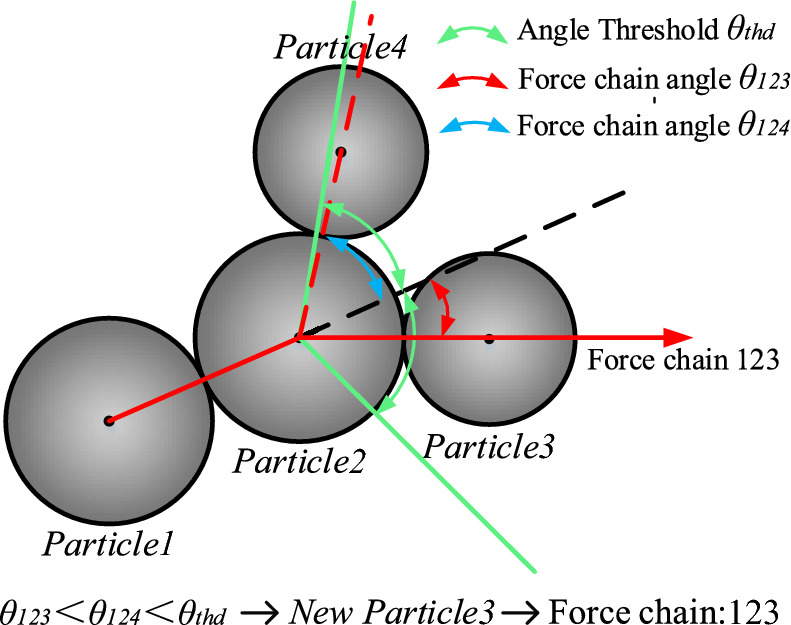
Identification of extension direction of force chain.

**Figure 23:**
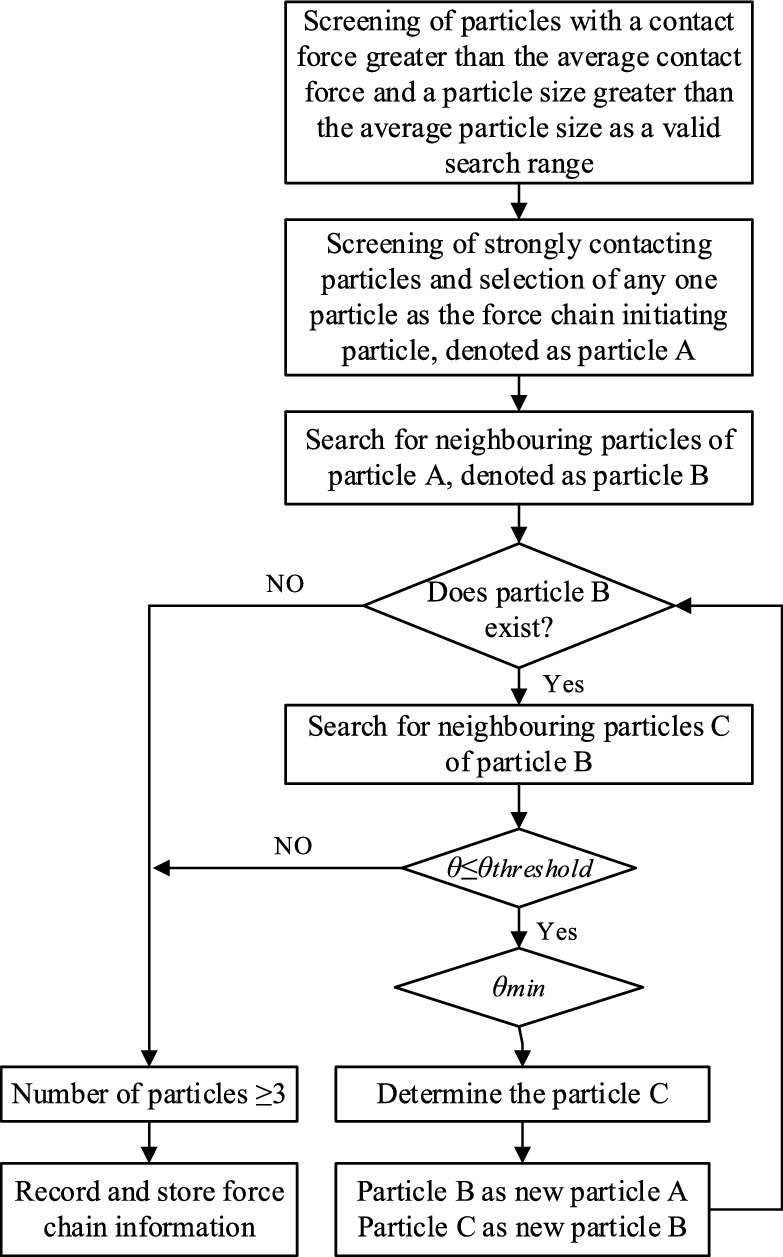
Force chain extraction program.

Among the above four conditions, the force criterion and particle size criterion serve as the basis for the other two criteria, the length criterion is the premise of the direction criterion, and whether or not a force chain can be formed or what kind of shape of force chain can be formed is mainly determined by the direction criterion. Figure 22 shows a schematic diagram of the extension direction of the force chain. When both particle3 and particle4 satisfy the force criterion and are within the range of the direction angle threshold, the smallest angle *θ*_*123*_ is taken as the extension direction of the force chain, and a force chain is formed as a result: *particle1*—*particle2*—*particle3.* Figure 23 shows the force chain extraction procedure, where all the information about the required particles is first extracted by the discrete element software, and then all the particles are screened using the criterion conditions, and after removing the duplicate force chains, the number and length of the force chains are finally stored.

### Validation of the applicability of the force chain into chain search procedure

**Figure 24:**
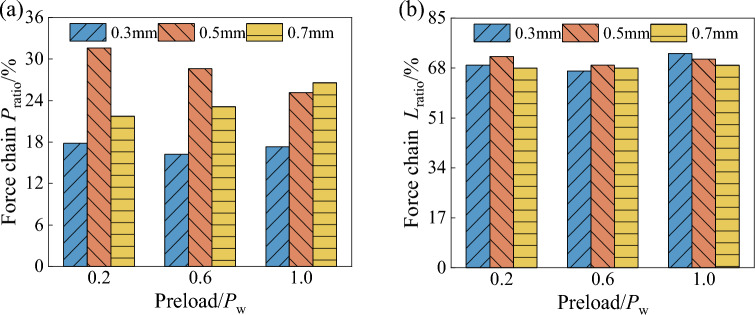
Force chain (**a**) composition ratios and (**b**) bearing ratios for different thicknesses of wet coal dust layer.

In order to verify the applicability of the force chain retrieval procedure proposed in this paper for wet coal dust layers, the force chain composition ratio and force chain bearing ratio are used as evaluation indexes. The force chain composition ratios and load-bearing ratios of different humid coal dust seams are calculated by Eqs. (24) and (25), as shown in Fig. 24.

24 $$ P_{ratio} = \frac{{Q_{{L{\text{n}}}} }}{{Q_{Tn} }} $$

25 $$ L_{ratio} = \frac{{F_{{L{\text{n}}}} }}{{F_{Tn} }} $$

where *P*_*ratio*_ is the ratio of force chain composition, *Q*_*Ln*_ is the total number of particles forming the force chain, and *Q*_*Tn*_ is the total number of particles in the wet coal dust layer. *L*_*ratio*_ is the ratio of force chain carrying, *F*_*Ln*_ is the sum of contact force of all force chains, and *F*_*Tn*_ is the sum of contact force of all particles in the wet coal dust layer.

As can be seen from Fig. 24, the ratio of force chain composition is stable at about 18% with increasing preload for a wet coal dust layer with a thickness of 0.3 mm. For the wet coal dust layer with a thickness of 0.5 mm, the ratio of force chain composition decreases with the increase of preloading force, which is caused by a large increase in the total number of particles due to particle fragmentation. The ratio of force chain formation is higher in the 0.5-mm-thick wet coal dust layer than in the other thicknesses of wet coal dust layer, up to about 30% of the number of particles forming force chains. For the 0.7 mm wet seam, the ratio of force chain formation increased from 20% to about 26% with the increase of preloading force. This result shows that not all the wet coal dust particles form a force chain, and the actual wet coal seam is loaded by a force chain structure composed of a small number of particles. The force chain of the wet coal dust layer carries about 70% of the external load, and the difference in the load carrying capacity of the force chain is relatively small in different thicknesses of the wet coal dust layer. The above results show that the force chain searched by the algorithm proposed in this paper can transfer most of the external loads, and the proposed force chain identification algorithm has good applicability.

## Quantitative analysis of force chain properties of a wet coal dust layer

**Figure 25:**
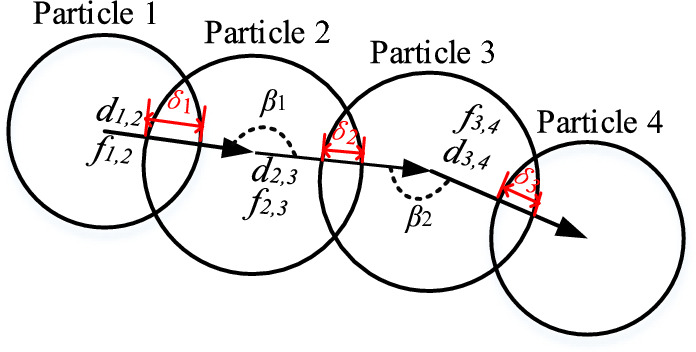
Schematic diagram of quantitative characteristics of the force chain.

In order to investigate the dynamic load-bearing process of the force chain in the wet coal dust layer, the force chain was characterised from the perspective of quantitative properties by defining the indexes such as the number of force chains, the length, the stiffness, and the yield degree, as shown in Fig. 25.

Let *N*_*i*_ be the number of particles on the ith force chain (i = 1,2*···*, *n*, *n* is the number of force chains in the wet coal dust layer), and the length and curvature of the *i*-th force chain are denoted as *l*_*i*_ and *C*_*i*_.

Length of one force chain *l*_*i*_:

26 $$ l_{i} = \sum\limits_{j = 1}^{{N_{i} - 1}} {d_{j,j + 1} } $$

where *d*_*j, j*+*1*_ denotes the distance between the *j*-th and (*j* + *1*)*-*th particle centre of mass.

Length of force chain in wet coal dust layer < *l*_*i*_ > :

27 $$ \left\langle {l_{i} } \right\rangle = \frac{{\sum\limits_{i = 1}^{N} {l_{i} } }}{N} $$

where *N* is the number of wet coal dust layer force chains.

Force chain curvature *C*_*i*_:

28 $$ C_{i} = \frac{{\sum\limits_{i = 1}^{n - 2} {\frac{{180^{^\circ } - \beta_{i} }}{{180^{^\circ } }}} }}{{l_{i} }} $$

where *β*_*i*_ is the angle between *d* and *d*. For example *β*_*1*_ is the angle between *d* and *d* in Fig. 22. Force chain curvature indicates that the force chain maintains a straight chain shape and can reflect the amount of stability of the force chain network^41^. A force chain with a large curvature is unstable in bearing and prone to fracture, while a smaller curvature is stable in bearing and less prone to fracture^42^.

Force chain curvature of wet coal dust layer < *C*_*i*_ > :

29 $$ \left\langle {C_{i} } \right\rangle = \frac{{\sum\limits_{i = 1}^{N} {C_{i} } }}{N} $$

The number of force chains, the length of force chains, the stiffness of force chains and the curvature of force chains mentioned later refer to the corresponding indexes of the wet coal dust layer.

### Calculate the three-body contact stiffness

**Figure 26:**
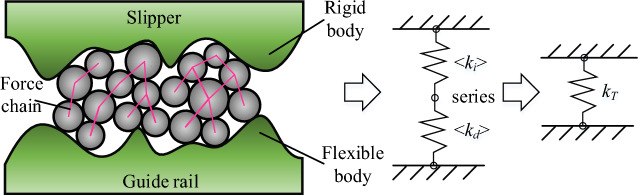
Simplified diagram of the stiffness of the three-body contact bond surface.

The stiffness at the three-body contact bonding surface mainly consists of two parts: the damp coal dust layer stiffness and the guideway stiffness. When the slide-shoe is pressed down to the stable moment, i.e., the bonding surface bearing is approximately equal to the external load, the wet coal dust layer particles are no longer broken. At this time, it is assumed that the wet coal dust fills the peaks and valleys of the slide boot and guide rail surface, and the area of the bonding surface is A_L_. The three-body contact bonding surface stiffness is regarded as the series connection of the wet coal dust layer stiffness and the micro-convex body stiffness of the guide rail surface. As shown in Fig. 26.

Normal stiffness of a force chain *K*_*i*_:

30 $$ K_{i} = \frac{{\sum\limits_{n = 1}^{Ni - 1} {f_{n} } }}{{\delta_{i} }} $$

31 $$ \delta_{i} = \sum\limits_{j = 1}^{{N_{i} - 1}} {R_{j} + R_{{j{ + 1}}} - |r_{j} { + }r_{{j{ + 1}}} |} $$

where *f*_*n*_ is the magnitude of the nth normal contact force of the *i-*th force chain, and the number of contact forces is *N*_*i*_*-*1; *δ*_*i*_ is the normal elastic deformation of the force chain; *R*_*j*_ and *R*_*j*+1_ are the radii of the two contacting particles in the *i-*th force chain; and *r*_*j*_ and *r*_*j*+1_ are the spherical centre position vectors of the two particles.

Different weights were given according to the length of each force chain, and then the stiffness values of each force chain were weighted and averaged. Finally, the normal contact stiffness per unit area of the wet coal dust layer is calculated.

Normal contact stiffness per unit area of wet coal dust layer < *k*_*i*_ > :

32 $$ \left\langle {K_{i} } \right\rangle = \frac{{k_{1} l_{1} + k_{2} l_{2} + ... + k_{N} l_{N} }}{{A_{L} (l_{1} + \, l_{2} + \, ... \, + \, l_{N} )}} $$

where A_L_ = L^2^ is the area of the bonding surface for the simulation setup.

Normal contact stiffness per unit area of guideway < *k*_*d*_ > :

33 $$ \left\langle {K_{d} } \right\rangle = \frac{{\left\langle {p_{d} } \right\rangle }}{{\left\langle {l_{d} } \right\rangle }} $$

where < *P*_*d*_ > is the contact pressure per unit area between the guide and wet coal dust, and < *l*_*d*_ > is the average normal deformation of the guide surface.

Total stiffness of three-body normal contact *K*_*T*_:

34 $$ K_{T} = \frac{1}{{\left( {{1 \mathord{\left/ {\vphantom {1 {\left\langle {k_{i} } \right\rangle }}} \right. \kern-0pt} {\left\langle {k_{i} } \right\rangle }} + {1 \mathord{\left/ {\vphantom {1 {\left\langle {k_{d} } \right\rangle }}} \right. \kern-0pt} {\left\langle {k_{d} } \right\rangle }}} \right)}} $$

### Influence of different porosities on three-body contact stiffness in a wet coal dust layer

The initial porosity of the wet coal dust layer is closely related to the force chain characteristics and structural evolution. The mechanical properties of the three-body contact bonding surface under the conditions of simulation (preload force *P*_*w*_ = 0.6Mpa; thickness of coal dust layer *H* = 0.5mm; porosity *ζ* = 0.4, 0.5, 0.6; water content *W* = 6%;) are simulated. The effect of different initial porosity on the bearing characteristics of the force chain is investigated, and then the effect of different coal dust layer thickness on the three-body contact stiffness is obtained.

**Figure 27:**
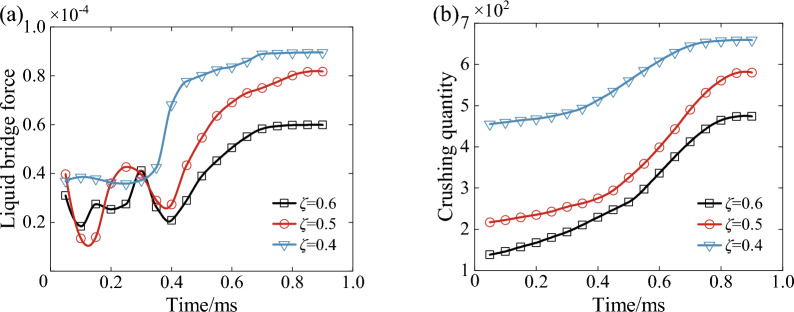
Variation of bonding surface parameters under different initial porosity conditions, (**a**) average liquid bridge force at the bonding surface, (**b**) number of particles fragmented.

Figure 27 shows the variation of three-body contact bonding surface parameters under different initial porosity conditions. From Fig. 27a, it can be seen that the average liquid bridge force of the wet coal dust layer fluctuates up and down without any regularity from 0 to 0.4 ms, and after 0.4 ms, it shows an overall increase firstly and then tends to be stable. And the average liquid bridge force becomes smaller with the increase of the initial porosity of the wet coal fly ash layer, which is because the initial porosity decreases the particle rearrangement of the wet coal fly ash layer decreases, the contact force between the particles is stable, and a large number of liquid bridges are formed in the wet coal fly ash layer. From Fig. 27b, it can be seen that the number of broken particles in the wet coal dust layer increases with the decrease of its initial porosity, and the smaller the porosity, the denser the contact of particles, the more concentrated the transfer of external load, the more the number of broken particles and the particles of the wet coal dust layer are no longer crushed after 0.8 ms.

**Figure 28:**
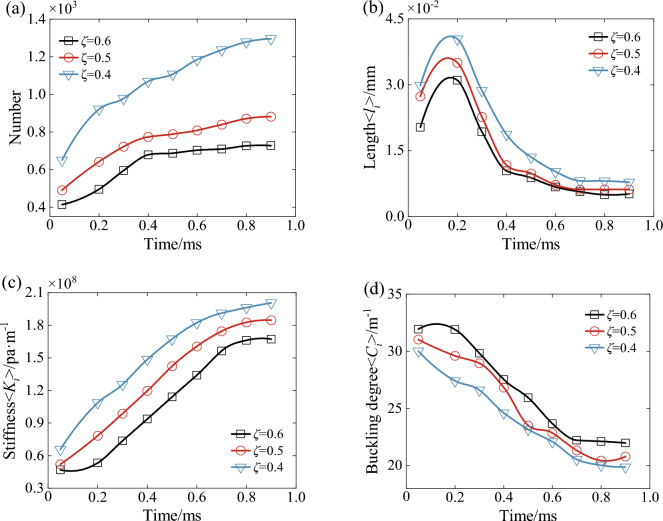
Force chain characteristics of wet coal dust layer under different initial porosity conditions, (**a**) number of force chains, (**b**) force chain length, (**c**) force chain stiffness, (**d**) force chain curvature.

Figure 28 shows the force chain characteristics of the wet coal dust layer under different porosity conditions. From the figure, it can be seen that in the process of downward pressure, the number of force chains and the stiffness of force chains in the wet coal dust layer firstly increased and then tended to be stable, while the bending degree of the force chains was opposite to its changing trend. At the same time, the length of the force chain increases and then decreases and finally tends to be stable, mainly because the loose wet coal dust layer is subjected to external load, and a large number of free particles inside it come into contact with each other, which leads to an increase in the length of the force chain. At 0.2 ms the force chain length reaches the maximum value of 3.1 × 10^−2^ mm, 3.5 × 10^−2^ mm and 4 × 10^−2^ mm.With the decrease of initial porosity, the number, length and stiffness of force chains in the wet coal dust layer increase significantly. At the moment of downward pressure to the stable moment, the number of force chains in the wet coal dust layer with initial porosity of 0.4 tends to be about 1295, the length of the force chains is 0.78 × 10^−2^ mm, and the stiffness of the force chains reaches the maximum value of 2.007 × 10^8^pa/m. The change trend of the bending degree of the force chains is opposite to that of the force chains, and the minimum bending degree decreases to 20°, which is a time when the wet coal dust layer is strong in bearing loads and has a high stability.

**Figure 29:**
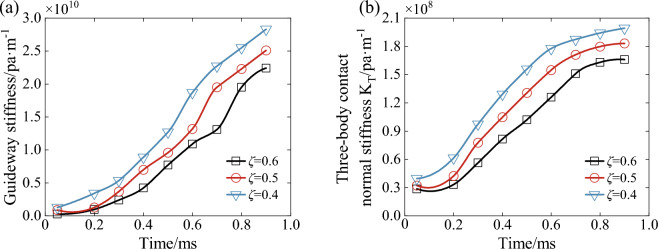
Variation of three-body contact bonding surface stiffness under different porosity conditions, (**a**) guideway surface stiffness, (**b**) three-body contact bonding surface stiffness.

Figure 29 shows the variation of the three-body contact bond surface stiffness under different porosity conditions. From the figure, it can be seen that the contact stiffness of the guideway surface gradually increases during the downward pressure process. And the trend of increase gradually slows down after 0.5 ms, which is because the contact area between the wet coal dust layer and the guide rail surface gradually tends to be stable after 0.5 ms. Even if some of the particles in contact with the guideway surface are crushed, the number of contact with the guideway surface is high. However, the contact area of large-size particles with the guideway surface is approximately unchanged compared with that before crushing. At the same time, as the wet coal dust layer porosity decreases, the guide rail surface contact stiffness increases. This is because when the porosity is small, the relative motion between the wet coal dust is small and the contact between the peaks and valleys of the guideway surface first appears in the contact steady state, and the maximum stiffness reaches 2.83 × 10^10^pa/m. As shown in Fig. 29b, the three-body contact bonding surface stiffness increases with the increase of time. Among them, before 0.5 ms, the contact stiffness of the guideway surface and the stiffness of the humid coal dust layer have a large effect on the three-body bonding surface stiffness at the same time. After 0.5 ms, the guideway surface stiffness is much larger than the wet coal dust layer stiffness, and the change trend of three-body contact stiffness is approximately the same as that of wet coal dust layer stiffness at this time.

The stiffness of the wet coal dust layer and the stiffness of the three-body contact bonding surface under different water content conditions were obtained by Eqs. (32–34) as shown in Table 4.

**Table 4:** Three-body contact stiffness under different initial porosity conditions.

Original porosity*ζ*	Wet coal layer stiffness $\left\langle {K_{i} } \right\rangle$ (pa/m)	Guideway stiffness $\left\langle {K_{d} } \right\rangle$ (pa/m)	Three-body contact normal stiffness *K*_T_ (pa/m)
0.4	2.007 × 10^8^	2.834 × 10^10^	1.993 × 10^8^
0.5	1.845 × 10^8^	2.513 × 10^10^	1.832 × 10^8^
0.6	1.671 × 10^8^	2.241 × 10^10^	1.659 × 10^8^

## Experimental verification of three-body contact stiffness at different porosities

This section focuses on verifying the three-body contact stiffness results for different porosities in the simulation by building a three-body contact model experimental rig containing wet coal dust.

**Figure 30:**
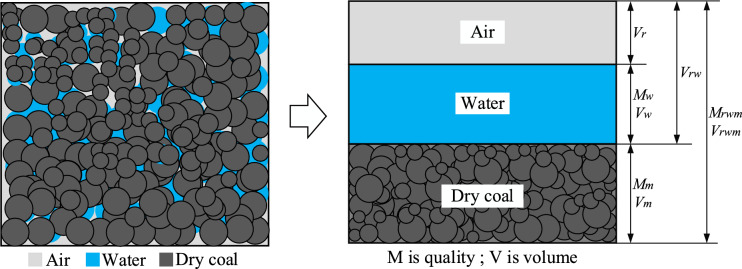
Schematic diagram of the composition of a wet coal dust layer.

The porosity of the wet coal dust layer consists of three components: solid, liquid and gas, and it is assumed that they exist in the form of each other as shown in Fig. 30, where M is the mass and V is the volume. Wet coal dust particle size extraction is described in detail in the previous section, where a rectangular container with dimensions of 40 × 100 × 0.5 mm was filled with wet coal dust, weighed and recorded. The water content was controlled to 6% by a water molecule detector, and the weight of the wet coal dust layer was adjusted to control the initial porosity to 0.4, 0.5, and 0.6, and the prepared specimens were labelled as *ζ*1, *ζ*2, and *ζ*3, respectively;

Porosity ratio *e*:

35 $$ e = \frac{{V_{r} + V_{w} }}{{V_{m} }} = \frac{{V_{rw} }}{{V_{m} }} $$

where *V*_*r*_ is the volume of air; *V*_*w*_ is the volume of water; *V*_*rw*_ is the total volume of air and water; *V*_*m*_ is the volume of dry coal dust.

Porosity *n*:

36 $$ n = \frac{{V_{r} + V_{w} }}{{V_{rwm} }} = \frac{{V_{rw} }}{{V_{rwm} }} \times 100\% $$

where *V*_*rwm*_ is the volume of wet coal dust layer.

The relationship is obtained from Eqs. (35) and (36):

37 $$ n = \frac{e}{1 + e} $$

Wet density *ρ*_*w*_:

38 $$ \rho_{w} = \frac{{M_{m} }}{{V_{rwm} }} = \frac{{M_{m} }}{{V_{r} + V_{w} }} = \frac{{M_{m} /V_{m} }}{{V_{m} /V_{m} + V_{rw} /V_{m} }} = \frac{{M_{m} /V_{m} }}{1 + e} $$

where *M*_*m*_ is the mass of dry coal dust.

Dry density *ρ*_*g*_:

39 $$ \rho_{g} = M_{m} /V_{m} $$

Wet and dry density relationships:

40 $$ \rho_{g} = \rho_{w} /(1 + W) $$

where *W* is the weture content of wet coal dust.

**Figure 31:**
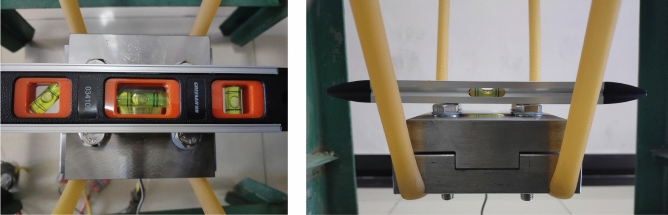
Level adjustment balance.

In this test, the three-body normal contact stiffness is obtained by measuring the intrinsic frequency of the three-body contact bonding surface, which consists of three parts: the sliding shoe specimen—the wet coal dust layer—the guide rail specimen. The three-body contact model was suspended in the air by rubber bands to simulate the three-body normal contact stiffness under the free boundary conditions, and the preload force of the three-body contact bonding surface was set to 0.6 MPa by adjusting the bolt preload force with an electronic torque wrench. Before applying external excitation to the slide boot with a force hammer, it was necessary to adjust the three-body contact model suspended by rubber bands by using the leveling device. Adjustment of the control level bubble is located in the middle of the balance scale position, indicating that the suspended three-body contact model is in a balanced position, as shown in Fig. 31.

**Figure 32:**
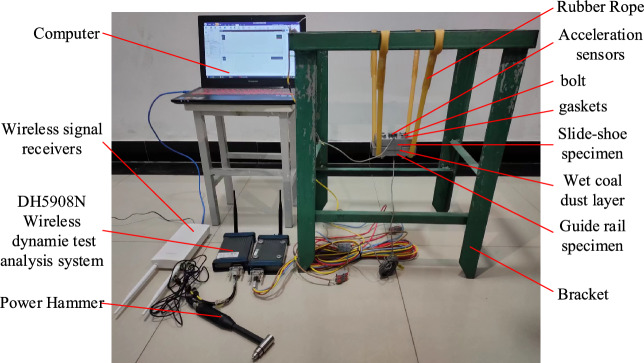
Pilot test site plan.

**Figure 33:**
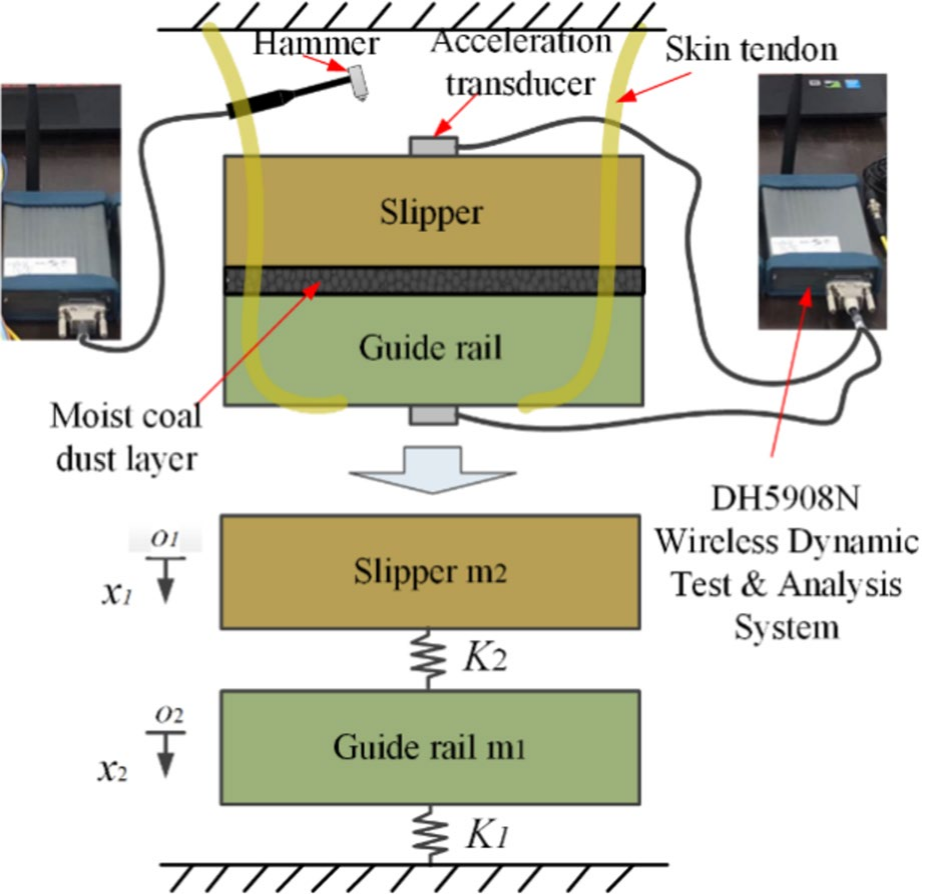
Experimental test schematic.

The physical diagram of the test device is shown in Fig. 32, the first step of the force hammer vertical upper plate to apply downward normal pulse excitation, through the sliding shoe and guideway acceleration sensor measurements to obtain real-time vibration data, from the wireless dynamic test and analysis system DH5908N transmitted to the wireless signal receiver, and finally transmitted to the computer and data processing. Figure 33 shows the schematic diagram of the test principle, and the three-body contact bonding surface is regarded as a spring damping unit. While the damping has a weak effect on the system's intrinsic frequency during the test, the three-body contact model is simplified to a two-degree-of-freedom undamped free vibration system^43^, and the masses of the upper and lower plates, m_1_ and m_2_, are obtained using electronic scales.

The vibration differential equation of the system can be obtained from Fig. 33:

41 $$ \left\{ \begin{gathered} m_{1} \ddot{x}_{1} + (k_{1} + k_{2} )x_{1} - k_{2} x_{2} = 0 \hfill \\ m_{2} \ddot{x}_{2} - k_{2} x_{1} + k_{1} x_{2} = 0 \hfill \\ \end{gathered} \right. $$

The characteristic equation of the system is:

42 $$ \begin{gathered} \left| {\begin{array}{*{20}c} {k_{11} - m_{1} \omega_{{\text{k}}}^{2} } & {k_{12} } \\ {k_{21} } & {k_{22} - m_{2} \omega_{k}^{2} } \\ \end{array} } \right| = 0\quad \hfill \\ k_{11} = k_{1} + k_{2} \quad k_{12} = k_{21} = - k_{2} \quad k_{22} = k_{2} \hfill \\ \end{gathered} $$

43 $$ \left\{ {\begin{array}{*{20}l} {\left. {\left( {k_{11} - m_{1} \omega_{k}^{2} } \right.} \right)\left( {k_{22} - m_{2} \omega_{k}^{2} } \right) - k_{12}^{2} = 0} \hfill \\ \begin{gathered} m_{1} m_{2} \left( {\omega_{k}^{2} } \right)^{2} - \left( {m_{1} k_{22} + m_{2} k_{11} } \right)\omega_{k}^{2} + k_{11} k_{22} - k_{12}^{2} = 0 \hfill \\ \omega_{k} = 2\pi f = \sqrt{\frac{K}{m}}  \hfill \\ K_{s} = \frac{K}{{A_{s} }} \hfill \\ \end{gathered} \hfill \\ \end{array} } \right. $$

where *ω*_*k*_ is the intrinsic frequency of the three-body system; K is the normal stiffness of the bonding surface; *A*_*s*_ = 4000 mm^2^ is the area of the bonding surface; *K*_*s*_ is the normal stiffness per unit area of the bonding surface; and m is the mass of the system.

The masses *m*_1_ and *m*_2_ of the sliding shoe and guideway were measured using an electronic scale, and the intrinsic frequency of the system, *ω*_*k*_, was measured from vibration tests. The three-body normal contact stiffness, *K*_*2*_, was obtained by solving Eq. (41) via MATLAB.

**Figure 34:**
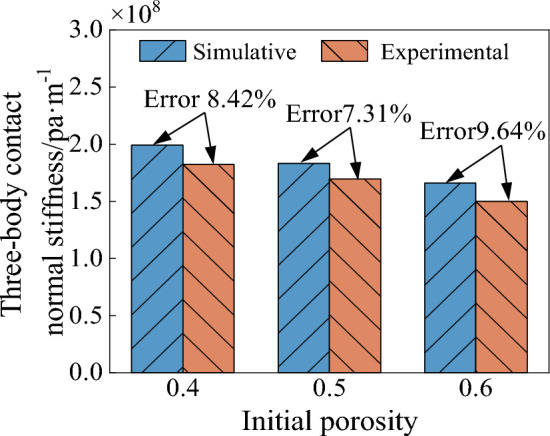
Experimental and simulated values of three-body normal contact stiffness with different initial porosity.

Figure 34 shows the simulated and experimental values of three-body normal contact stiffness for different initial porosities. From the figure, it can be seen that the three-body contact normal stiffness decreases with the increase of initial porosity. The maximum error between simulated and experimental values are 8.42%, 7.31% and 9.64% respectively. Thus, the accuracy of the force chain extraction procedure and the quantification of the force chain to calculate the stiffness in this paper are demonstrated.

Reasons that may lead to errors between simulation and test include: (1) When obtaining the surface morphology of the guideway on the bonding surface, there may be errors in the rough surface data obtained due to the influence of environmental factors. (2) Spherical particles are used to represent the shape of coal dust in the simulation process, which is different from the actual shape, which may result in the wet coal dust layer not reflecting the actual load-bearing state well. The above factors may have an impact on the simulation results, which may lead to a certain deviation between the simulation and the experimental results.

## Conclusions

In this paper, for the three-body contact stiffness problem of the slide-shoe and guideway containing wet coal dust interface, the coupling method of finite element and discrete element is used, and the discrete nature of the particles at the wet coal dust interface and the deformation of the contact surface of the slide-shoe and guideway are considered comprehensively. A real three-body contact simulation model containing a wet coal dust interface is constructed, and the force chain formation criterion is formulated according to the bearing characteristics of the wet coal dust layer. Finally, the three-body normal contact stiffness under different initial porosities is obtained by quantitative calculation of the force chain and verified experimentally. The corresponding conclusions are as follows.

1. Wet coal dust layer greater than the average pressure contact force of the particles stored more than 90% of the cumulative energy of elasticity, and its performance of the various anisotropic contact force and the overall anisotropy of the wet coal dust layer is approximately the same as that of the wet coal dust layer, the average contact force of the wet coal dust layer can be used as the threshold value of the contact force of the force chain.
2. The wet coal dust layer is larger than the average particle size particles bearing ratio is greater than 90%, in order to improve the retrieval efficiency of the force chain, the average particle size of the wet coal dust layer as can be used as the force chain particle size threshold.
3. Particle crushing in the wet coal dust layer produces significant grading effects, and the force chain angle threshold is obtained by analysing the adjacent contact angle, which is determined by the particle coordination number.
4. The number of broken particles in the wet coal dust layer increases with the decrease of its initial porosity, the smaller the porosity, the denser the contact of particles, the more concentrated the transfer of external loads, and the more the number of broken particles.
5. The number of force chains and the stiffness of force chains in the humid coal dust layer firstly increase and then tend to be stable, while the curvature of force chains is opposite to its trend. At the same time, the length of force chain firstly increases and then decreases and finally tends to be stable, and the maximum value of force chain length reaches 3.1 × 10^−2^ mm, 3.5 × 10^−2^ mm and 4 × 10^−2^ mm at 0.2 ms.
6. With the decrease of initial porosity, the number, length and stiffness of force chains in the wet coal dust layer increase significantly. At the moment of downward pressure to the stable moment, the number of force chains in the wet coal dust layer with initial porosity of 0.4 tends to be 1300, the length of force chain is 0.78 × 10^−2^ mm, and the stiffness of the force chain reaches the maximum value of 2.007 × 10^8^pa/m. And the trend of change of bending degree of the force chain is opposite to that of the force chain, and its minimum bending degree decreases to 20°, at this time, the wet coal dust layer is strong in load bearing and high in stability.
7. The maximum errors between the simulated and experimental values of the normal stiffness of the three-body contact under different initial porosity conditions are 8.29%, 7.31%, and 9.64%, respectively, which proves the accuracy of the force-chain formation criterion as well as the method of quantitatively calculating the stiffness of the three-body contact with the force chain.

## Data Availability

The datasets used and/or analysed during the current study available from the corresponding author on reasonable request.
